# Identification of prognostic immune-related gene signature associated with tumor microenvironment of colorectal cancer

**DOI:** 10.1186/s12885-021-08629-3

**Published:** 2021-08-08

**Authors:** Yuanyuan Wang, Wei Li, Xiaojing Jin, Xia Jiang, Shang Guo, Fei Xu, Xingkai Su, Guiqi Wang, Zengren Zhao, Xiaosong Gu

**Affiliations:** 1 Academy of Medical Engineering and Translational Medicine, Tianjin University, Tianjin, 300072 China; 2 Department of General Surgery, Hebei Key Laboratory of Colorectal Cancer Precision Diagnosis and Treatment, The First Hospital of Hebei Medical University, Donggang Road 89, Shijiazhuang, 050031 Hebei China; 3 Departments of Emergency, The First Hospital of Hebei Medical University, Shijiazhuang, Hebei China

**Keywords:** Colorectal cancer, Immune-related gene signature, Tumor microenvironment, TCGA, GEO, Prognosis

## Abstract

**Background:**

The tumor microenvironment (TME) has significantly correlation with tumor occurrence and prognosis. Our study aimed to identify the prognostic immune-related genes (IRGs)in the tumor microenvironment of colorectal cancer (CRC).

**Methods:**

Transcriptome and clinical data of CRC cases were downloaded from TCGA and GEO databases. Stromal score, immune score, and tumor purity were calculated by the ESTIMATE algorithm. Based on the scores, we divided CRC patients from the TCGA database into low and high groups, and the differentially expressed genes (DEGs) were identified. Immune-related genes (IRGs) were selected by venn plots. To explore underlying pathways, protein-protein interaction (PPI) networks and functional enrichment analysis were used. After utilizing LASSO Cox regression analysis, we finally established a multi-IRGs signature for predicting the prognosis of CRC patients. A nomogram consists of the thirteen-IRGs signature and clinical parameters was developed to predict the overall survival (OS). We investigated the association between prognostic validated IRGs and immune infiltrates by TIMER database.

**Results:**

Gene expression profiles and clinical information of 1635 CRC patients were collected from the TCGA and GEO databases. Higher stromal score, immune score and lower tumor purity were observed positive correlation with tumor stage and poor OS. Based on stromal score, immune score and tumor purity, 1517 DEGs, 1296 DEGs, and 1892 DEGs were identified respectively. The 948 IRGs were screened by venn plots. A thirteen-IRGs signature was constructed for predicting survival of CRC patients. Nomogram with a C-index of 0.769 (95%CI, 0.717–0.821) was developed to predict survival of CRC patients by integrating clinical parameters and thirteen-IRGs signature. The AUC for 1-, 3-, and 5-year OS were 0.789, 0.783 and 0.790, respectively. Results from TIMER database revealed that CD1B, GPX3 and IDO1 were significantly related with immune infiltrates.

**Conclusions:**

In this study, we established a novel thirteen immune-related genes signature that may serve as a validated prognostic predictor for CRC patients, thus will be conducive to individualized treatment decisions.

**Supplementary Information:**

The online version contains supplementary material available at 10.1186/s12885-021-08629-3.

## Background

Colorectal cancer (CRC) is ranked as the third most common cause of cancer-related mortality globally. In the gastrointestinal tract, the incidence of CRC is one of the most frequently diagnosed [1]. The heterogeneity of CRC exists with differences in molecular pathogenesis, clinical features, and prognosis [2]. A significant number of early-stage CRC patients successfully undergo aggressive surgical removal of the primary tumors [2]. However, among stage II/III patients, the association of the recurrence with metastases has been evident [3]. Stage IV patients with high risks of metastatic relapses receive standardized cytotoxic chemotherapy [4]. Generally, the treatment of CRC involves a combination of one or more cancer drugs in their regimens, such as 5-fluorouracil (5-FU), folinic acid, irinotecan and oxaliplatin [5]. But in the presence of metastases, new adjuvant chemotherapy may not produce successful results as it could lead to cancer progression and the patient’s resistance to the drug [6].

Although several high-risk pathological characteristics, such as venous invasion or serosal involvement, are now recognised as important determinants of survival, particularly in node negative disease [7, 8]. However, it has become evident that the profiles of other hosts and tumors could also impact the clinical prognosis. Furthermore, the fundamental characteristics of tumor cells and mechanisms within the tumor microenvironment (TME), such as tumor-infiltrating immune cells (TIICs) [9] and stromal cells linked to the tumor, affect the progression of CRC and the clinical outcomes [10].

In epithelial cancer cells, the increased presence of mesenchymal genes leads to the recommendation that the poor prognosis of CRC patients is associated with the epithelial-to-mesenchymal transition [11]. However, the transcriptome of the tissue that contains the tumor characterizes the expression profiling of mesenchymal cells that establish the TME and the epithelial cancer cells [12, 13]. The stroma or TME is comprised of the structural and functional features of the connective tissue in homeostasis and pathological angiogenesis in wound healing and illness. Furthermore, the stroma is made up of lymph and blood vessels, inflammatory/immune cells, extracellular matrix, and fibroblasts. The cancer cells alter the dynamic and multifaceted structure of the stroma, which eventually affects the progression of malignant cells [14].

The two main classifications of non-tumor mechanisms are the stromal and immune cells in the TME. These play a significant role when diagnosing, making a prognosis for, and assessing tumors. In the recent decade, researchers have created several algorithms to forecast the purity of tumors in different types of cancer. These algorithms are grounded on the precise gene signature of immune cells and/or stromal cells [15, 16]. An example is the Estimation of STromal and Immune cells in MAlignant Tumor tissues using Expression data (ESTIMATE), which is an algorithm designed by Yoshihara K et al. [17]. This algorithm calculates both stromal score and immune score to forecast the presence of infiltrating non-tumor cells by analyzing the signatures of specific genes.

After the founding of the ESTIMATE logarithm, research studies have applied it to prove the efficacy of big-data algorithms on evaluating prostate cancer [18], glioblastoma [19], and cutaneous melanoma [20]. Nevertheless, researchers also need to extensively study the value of the ESTIMATE when evaluating stromal and/or immune scores of CRC. In this literature, we have pioneered the extraction of the list that itemizes how the TME-associated genes forecast poor prognosis amongst CRC patients. We completed this by using the data of CRC cohorts from Gene Expression Omnibus (GEO) and The Cancer Genome Atlas (TCGA).

## Methods

### Gene expression data sets

Our study utilized publicly available data sets. We collected gene expression profiles and identified the equivalent material on the prognosis, as well as the tumor and normal tissues of CRC patients. We obtained the data from the TCGA (https://tcga-data.nci.nih.gov/tcga/) and the GEO (https://www.ncbi.nlm.nih.gov/geo/) web sites, which were uploaded up to 31 March 2019. Moreover, we excluded duplications and datasets that have sample sizes of less than 50 (*N* < 50). Then, we organized the clinical data and expression profiles of each sample manually. Our inclusion criteria indicate that we include diagnosed CRC patients who have available clinicopathological and survival information. Therefore, six datasets were included in our study (GSE12945, GSE39582, GSE41258, GSE72970, GSE103479 and TCGA) [21–26]. Stromal scores, immune scores and tumor purity were calculated by the ESTIMATE algorithm [17]. For the succeeding genomic analysis, we used the TCGA dataset. To authenticate the prognosis of genetic information recognized by the TCGA analysis, we selected the largest CRC dataset GSE39582 as the validation cohort from the GEO database. This study complied with the TCGA and GEO approved publication guidelines. Also, we sourced the data from both databases. Thus, this research did not require the approval of the ethics committee.

### Identification of immune-related genes (IRGs)

Based on the ESTIMATE results, we classified the sample groups of the stromal scores, immune scores and tumor purity into high and low to choose the intersection genes. This study analyzed the data using the R package limma [27]. The cutoff values were established at FDR < 0.05 and Fold change > 2 to filter the differentially expressed genes. Finally, we obtained intersect genes among stromal scores, immune scores and tumor purity as immune-related genes (IRGs).

### Functional enrichment of IRGs

To complete the functional enrichment analysis of the IRGs and classify the GO categories according to the molecular functions (MF), the biological processes (BP), or the cellular components (CC), we used a R package called “clusterprofiler” [28]. Additionally, we utilized it to make a pathway enrichment analysis while referring to the pathways of Kyoto Encyclopedia of Genes and Genomes (KEGG). Finally, the cut-off value was set at a false discovery rate (FDR) < 0.05.

### Protein-protein interaction (PPI) network

The STRING database [29] produced the PPI network that the Cytoscape software rebuilt [30]. For further examination, this study only included individual networks with 10 or more nodes and excluded those with less than 10. The connectivity degree of each network node was calculated, and then, we searched for the clusters according to their typology to trace densely connected regions using the Molecular COmplex DEtection (MCODE).

### Establishment of the IRGs signature for CRC

To investigate the link between IRGs and the prognosis of CRC patients, we applied the univariate Cox regression analysis. In this analysis, the statistical significance was established at *p* < 0.05. We selected a panel of genes according to these outcomes through the LASSO Cox regression analysis that used the R package “glmnet”. Then, we set up a multigene signature to forecast the prognosis of these patients. By conducting cross-validations 1000 times and adopting the best penalty parameter λ value’s one standard error, we established the most simplified (smallest perimeter) model of immune gene expression signatures. For each patient, the sum of the corresponding coefficients and products of each gene expression level determined the risk score formula. To verify the efficiency of the signature constructed by the TCGA cohort, we validated the results in the GEO cohort (GSE39582).

### TIMER database analysis

To methodically analyses the immune infiltrates in different cancers, TIMER is a wide-ranging resource (https://cistrome.shinyapps.io/timer/) [31]. This algorithm uses a statistical deconvolution method, which was recently made available to infer the TIICs based on the gene expression profiles [15]. Moreover, TIMER covers 32 types of cancer and contains 10,897 samples from TCGA to approximate the abundance of immune-infiltrating. Our analysis encompassed the identification of prognostic immune-related genes (IRGs) in CRC and its correlation with the abundance of immune-infiltrating. These infiltrates include macrophages, neutrophils, B cells, CD8^+^ T cells, CD4^+^ T cells and dendritic cells (DC) through gene modules. On the left-most panel, the graph illustrates the gene expression profiles against tumor purity [32].

### Statistical analysis

Results were showed as mean ± standard deviation (SD). To contrast the immune score, stromal score and tumor purity in different groups, we used the Kruskal-Wallis test of variance. The cutoff value of high and lower groups of immune score, stromal score and tumor purity were calculated by X-tile [33]. We calculated the OS using the Kaplan-Meier method, and the statistical significance was determined by the log-rank test. The specificity and sensitivity of survival prediction according to the determined risk score were obtained by time-dependent receiver operating characteristic (ROC) curves, with AUC values quantified with the survivalROC package. This paper also executed the univariate and multivariate analyses of OS to distinguish the prognostic determinants of CRC patients from the TCGA and GSE39582 datasets. Furthermore, using the R package “rms”, we established a nomogram according to the TCGA CRC cohort and incorporated different prognostic factors. Moreover, we analyzed its performance through the C-index, calibration plots and decision curve analysis (DCA). Additionally, this study utilized the R software version 3.6.0 to conduct all of the statistical analyses (http://www.rproject.org/). Finally, we set the statistical significance at a two-sided *P* < 0.05.

## Results

### Association of stromal scores, immune scores and tumor purity with CRC prognosis

Stromal scores, immune scores and tumor purity were significantly associated with CRC clinical stages and prognosis. Using a variety of technologies, we obtained the genomic profiles. The stromal and immune scores, as well as the tumor purity, were generated through the uniform algorithm. The results did not reveal any obvious cohort-bias clustering. Hence, we merged the groups from TCGA and GEO and further investigated whether there were stromal scores, immune scores and tumor purity statistically correlated with CRC clinical stages and prognosis. In the present study, we downloaded the gene expression profiles and clinical data of 1635 CRC patients from the TCGA and GEO databases. Patients were 66.06 ± 12.95 years old, with 882 males (53.9%) and 753 (46.1%) females.

**Fig. 1:**
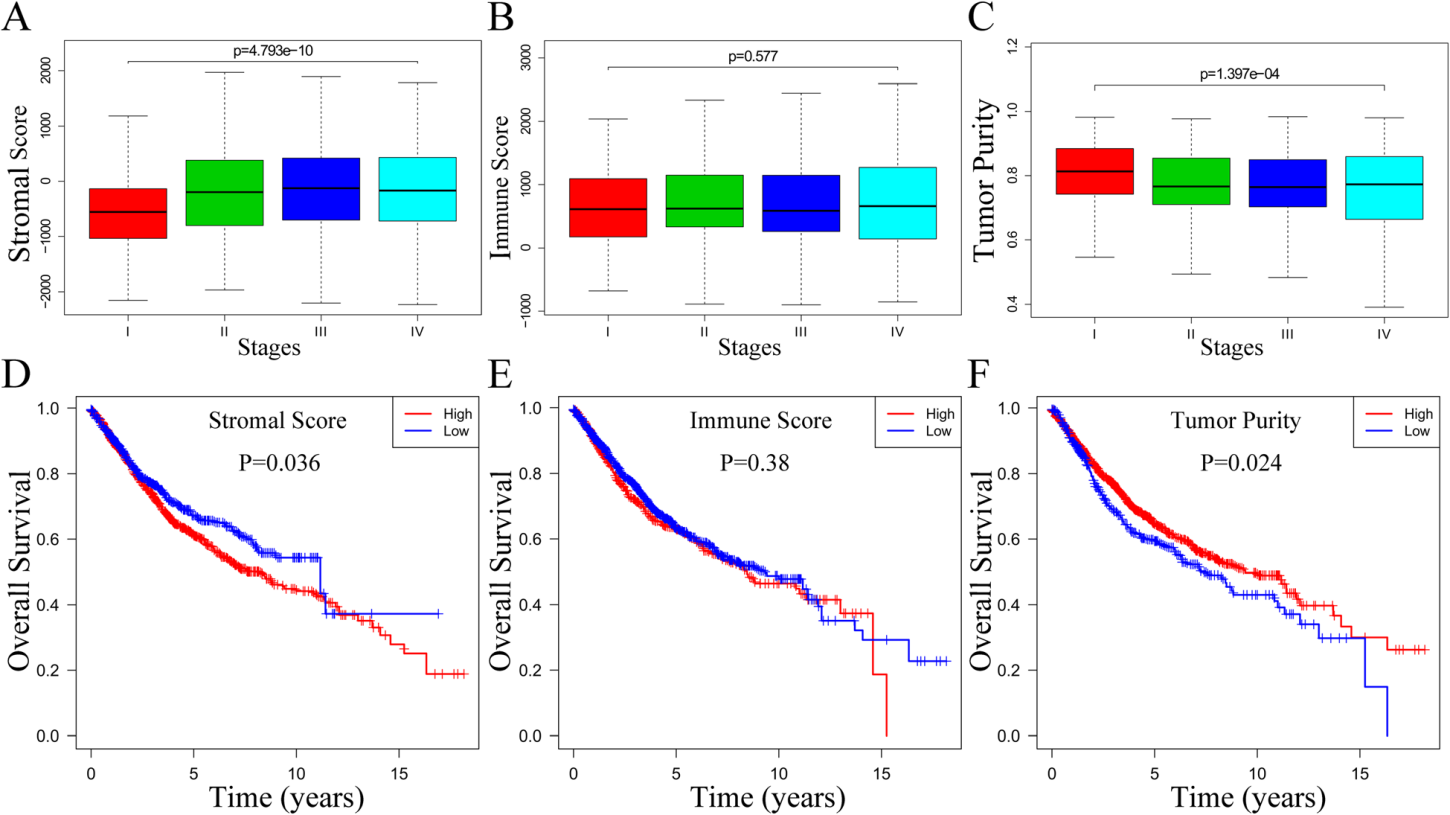
Immune score, stromal score and tumor purity are associated with tumor stages and patients’ OS. **A**, Distribution of stromal scores of tumor stages. **B**, Distribution of immune scores of tumor stages. **C**, Distribution of tumor purity of tumor stages. **D**, Comparison of overall survival between high and low stromal scores groups. **E**, Comparison of overall survival between high and low immune scores groups. **F**, Comparison of overall survival between high and low tumor purity groups

For CRC, TNM stages are valuable prognostic indicators. We included them in the analysis. The stromal scores ranged from − 2232.54 to 2193.09, immune scores were distributed between − 899.57 and 3202.84, and tumor purity ranged from 0.28 to 0.98, respectively (Data were calculated by ESTIMATE algorithm). Figure 1 illustrates that across different stages, the distribution of stromal scores differed (*p* = 4.79e-10) while the distribution of immune scores had no variation (*p* = 0.58). Figure 1 show that tumor purity (*p* = 1.40e-04) were inversely associated with different stages.

To study the possible correlation of the prognosis with stromal score, as well as immune score and tumor purity, we classified the CRC patients into high and lower groups. For this purpose, we created the X-tile and Kaplan-Meier survival curves. The results suggest that the stromal scores were positive correlation with the OS (*p* = 0.036) is statistically significant. Also, the immune scores are positively associated with OS, but it was not statistically significant (*p* = 0.38). Conversely, tumor purity was significantly negative association with OS (*p* = 0.024) (Fig. 1).

### Identification of DEGs

**Fig. 2:**
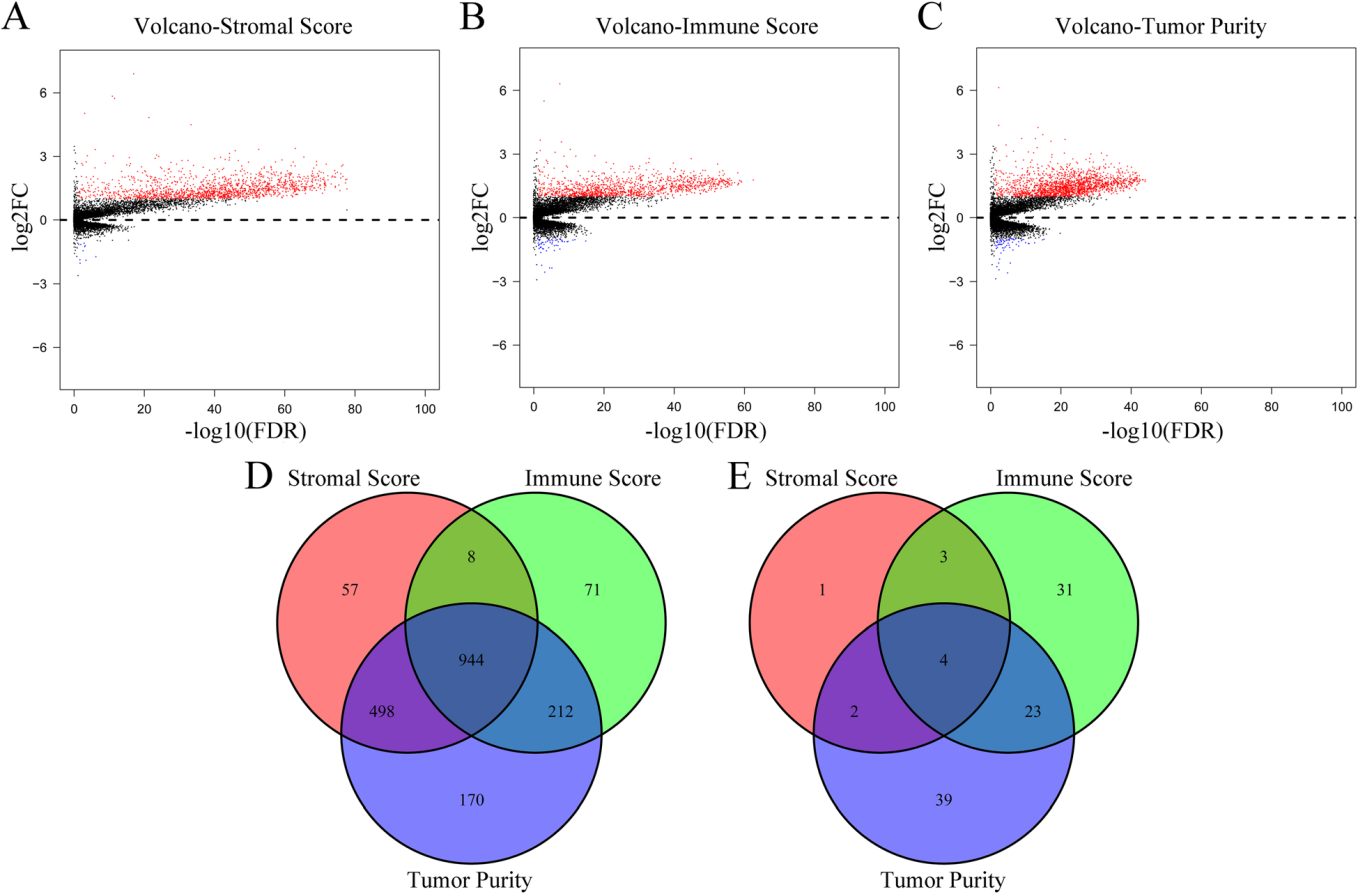
Comparison of gene expression profiles with stromal score, immune score and tumor purity. **A**, Volcano plots of the DEGs based on stromal score. **B**, Volcano plots of the DEGs based on immune scores. **C**, Volcano plots of the DEGs based on tumor purity. **D**, Venn diagrams shown the number of upregulated DEGs in stromal score, immune score and tumor purity groups. E, Venn diagrams shown the number of downregulated DEGs in stromal score, immune score and tumor purity groups

To uncover the correlation of gene expression profiles with stromal scores, immune scores and tumor purity, we analyzed RNAseq data of all 611 CRC cases obtained from the TCGA database. Figure 2, the volcano plots highlight unique gene expression profiles of cases that we classified under high/low stromal scores, immune scores, and tumor purity groups. We based our comparative analyses on three factors: first, stromal scores through the upregulation of 1507 genes and downregulation of 10 genes in the high stromal score group; second, immune scores through the upregulation of 1235 genes and downregulation of 61 genes in the high and low groups; and third, tumor purity group through the upregulation of 1824 genes and downregulation of 68 genes in the low score group. Furthermore, Fig. 2 illustrates the IRGs were the upregulated or downregulated intersection genes in the low tumor purity group and high stromal or immune groups (944 upregulated genes and 4 downregulated genes).

### Functional analysis of IRGs

**Fig. 3:**
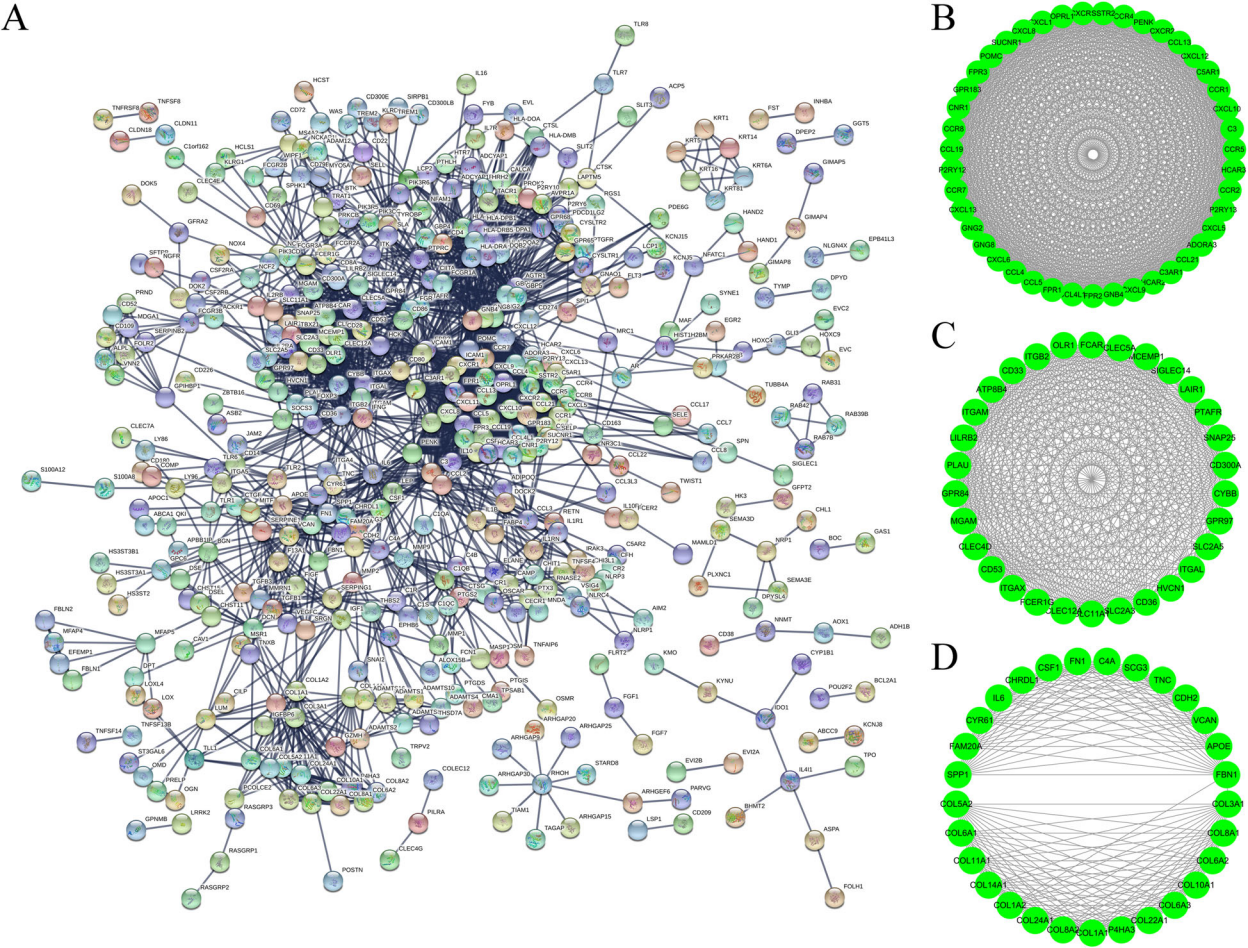
PPI network of immune-related genes obtained from the STRING database. **A**, PPI network of IRGs. **B**-**D**, The PPI networks of top 3 modules

Using the STRING tool that included 2834 edges and 471 nodes, we generated PPI networks to study the interactions among the identified immune-related genes (Fig. 3A). For further analysis, we chose three modules with at least 20 nodes. As shown in module A (Fig. 3B), the network had formations of 903 edges and 43 nodes. Each gene in this model was associated with 42 other genes, indicating that this module might be the core sub-network of PPI. In module B (Fig. 3C), there were 395 edges and 43 nodes that had numerous genes in the middle that were critical to immune response. These included OLR1, ITGB2, SLC2A3, ITGAM, and ATP8B4. In module C (Fig. 3D), there were 198 edges and 29 nodes where the higher degree values of connectivity in FBN1, COL3A1, and COL1A2 proved their existence as the core genes in the module.

**Fig. 4:**
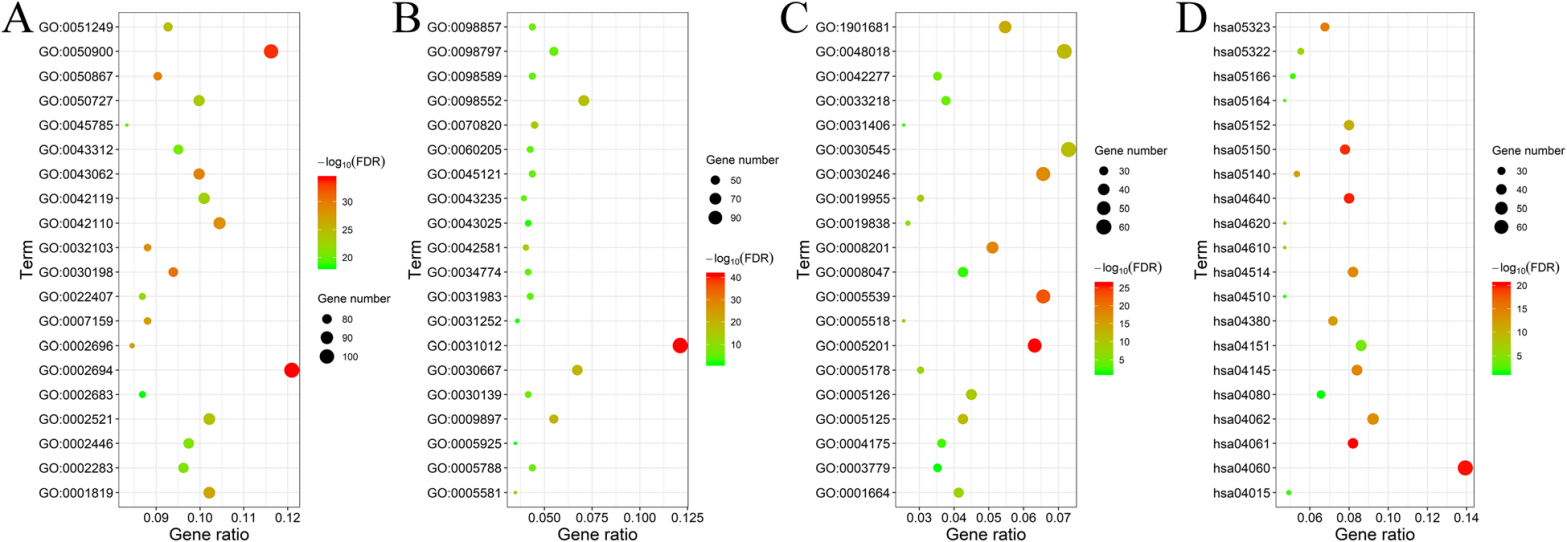
GO enrichment and KEGG pathway functional enrichment analyses of the IRGs. **A**, The biological process (BP) of GO classification. **B**, The cell component (CC) of GO classification. **C**, The molecular function (MF) of GO classification. **D**, KEGG pathway functional classification and annotation

The clustering of these enriched functional-related genes is associated with the immune response, which is in line with the PPI network analysis. Furthermore, we identified statistically significant GO terms, such as the total of 1325 for biological processes, 83 for molecular function, and 77 for the cellular component (FDR < 0.05). GO terms (Table S1) included regulation of leukocyte activation, leukocyte migration and T cell activation (Fig. 4A), extracellular matrix and side of membrane (Fig. 4B), and receptor regulator activity (Fig. 4C). Additionally, the KEGG analysis (Fig. 4D) generated pathways that were all linked with the immune response.

### Construction and validation of the IRGs-based signature

**Fig. 5:**
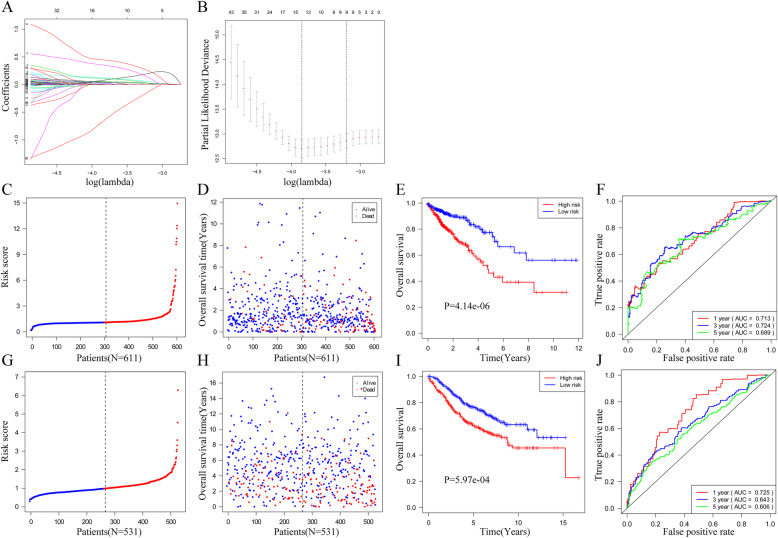
Construction and validation of the thirteen-IRGs signature. **A**, LASSO coefficient profiles of the IRGs associated with the overall survival of CRC. **B**, Partial likelihood deviance for the LASSO coefficient profiles. **C**, The risk score distribution on the basis of the thirteen-IRGs signature in TCGA cohort. **D**, The survival status of 611 patients with CRC in high- and low-risk groups. **E**, Kaplan–Meier curves for overall survival prediction by the thirteen-IRGs signature in TCGA cohort. **F**, Time-dependent ROC curves for overall survival prediction by the thirteen-IRGs signature in TCGA cohort. **G**, The risk score distribution on the basis of the thirteen-IRGs signature in GSE39582 cohort. **H**, The survival status of 531 patients with CRC in high- and low-risk groups. **I**, Kaplan–Meier curves for overall survival prediction by the thirteen-IRGs signature in GSE3958 cohort. **J**, Time-dependent ROC curves for overall survival prediction by the thirteen-IRGs signature in GSE3958 cohort

In the context of this study’s TCGA CRC patients, it utilized the univariate Cox models to investigate the association between the expression levels of their IRGs and their OS, 171 IRGs were found to have significant relationship with OS. Then, based on those 171 IRGs, we utilized the LASSO Cox regression model to build a prognostic signature in the TCGA dataset (Fig. 5A and B). Finally, a prognostic signature including thirteen IRGs was constructed. Using the coefficients derived from the LASSO Cox regression model, we constructed a formula to calculate risk score for each patient. This score is based on their personalized expression levels of the thirteen IRGs. The formula is as follows: risk score = (0.1217 × expression of A2ML1) + (0.03442 × expression of CALB2) + (− 0.6693 × expression of CD1B) + (0.04806 × expression of COL22A1) + (0.4471 × expression of FCRL2) + (0.00069 × expression of GPX3) + (0.05368 × expression of HAND1) + (0.0023 × expression of IDO1) + (0.006 × expression of LAMP5) + (0.07625 × expression of MAP2) + (0.02431 × expression of MMRN1) + (0.1085 × expression of NKAIN4) + (0.3541 × expression of VAX2). Patients were classified into low- and high-risk groups according to a cutoff risk score of 1.096 (Fig. 5C and D). Figure 5E demonstrates that the high-risk group had substantially lower levels of OS than in the low-risk at *p* < 0.0001. For 1-, 3-, and 5-year OS, the area under the ROC curve (AUC) was 0.713, 0.724, and 0.689, respectively (Fig. 5F). The GSE39582 cohort verified the prognostic model (*N* = 531). Patients were classified into low- and high-risk groups according to an ideal cutoff risk score of 0.973 (Fig. 5G and H). Figure 5I illustrates that the high-risk group had substantially lower levels of OS than in the low-risk at *p* = 5.97e-04. For 1-, 3-, and 5-year OS, the AUC was 0.725, 0.643, and 0.606, respectively (Fig. 5J).

### Independent prognostic role of the thirteen-IRGs signature

**Fig. 6:**
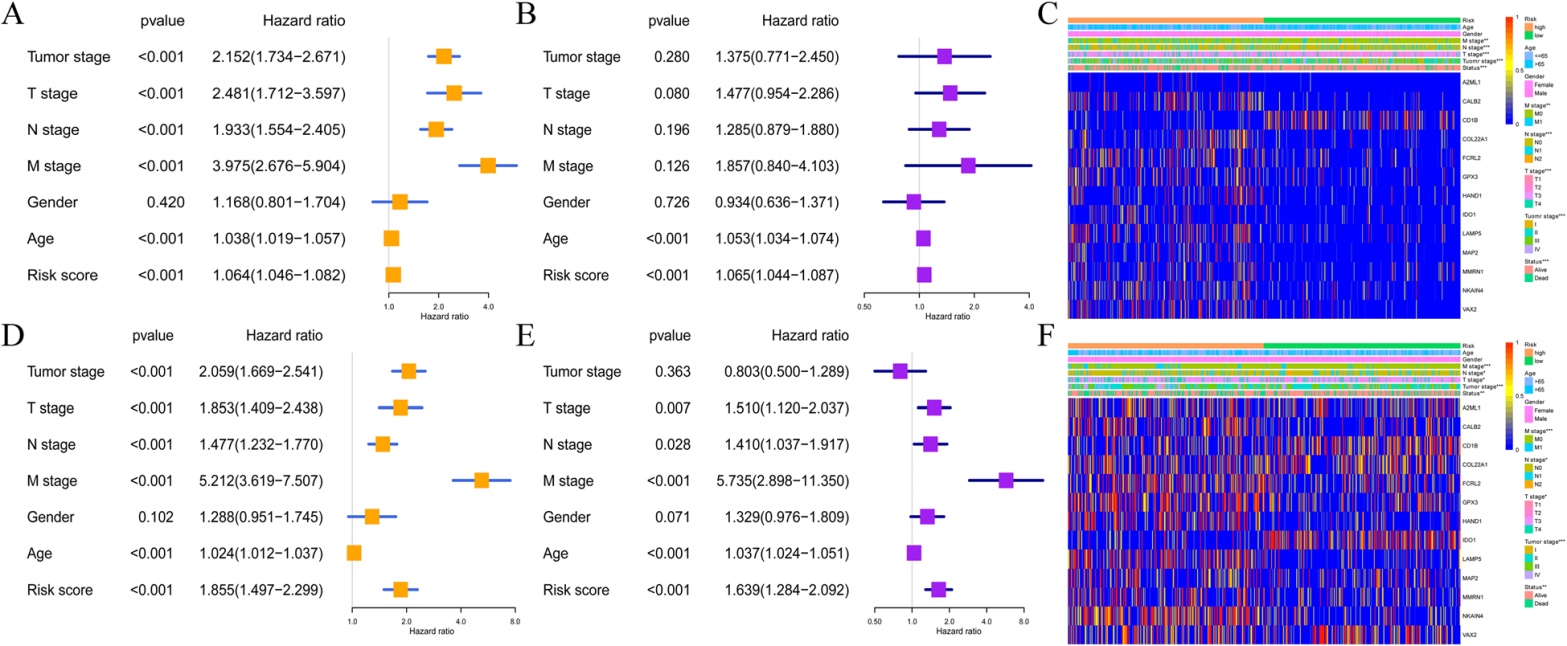
Independent prognostic role of the thirteen-IRGs signature. **A**, Univariate and analyses of clinicopathological features associated with overall survival in TCGA cohort. **B**, Multivariate analyses of clinicopathological features associated with overall survival in TCGA cohort. **C**, Association of the thirteen-IRGs signature with clinical parameters in TCGA cohort. **D**, Univariate and analyses of clinicopathological features associated with overall survival in GSE39582 cohort. **E**, Multivariate analyses of clinicopathological features associated with overall survival in GSE39582 cohort. **F**, Association of the thirteen-IRGs signature with clinical parameters in GSE39582 cohort

The univariate and multivariate Cox regression analyses were executed in both GEO and TCGA datasets through the adjustment of clinicopathological features like the T stage, N stage, M stage, and tumor stage, as well as the age and gender. This process aimed to assess if the signature-based risk score of the thirteen-IRGs was an independent prognosis factor for OS, which the results confirmed as true (Fig. 6A, B, D and E). We verified the clinical significance of the thirteen-IRGs signature using the Chi-square test to ascertain the signature’s association with the clinical parameters. In the TCGA cohort, significant correlations were found between the higher-risk score and tumor stage (*p* < 0.001), M stage (*p* < 0.01), N stage (*p* < 0.001), and T stage (*p* < 0.001) (Fig. 6C). However, no significant difference was found in gender and age. Comparable outcomes were observed in the validation cohort of GSE39582 (Fig. 6F).

To further analyze the hierarchical efficacy of the thirteen-IRGs signature, stage I, II and stage III, IV colorectal cancer patients in the TCGA data set were divided into high-risk and low-risk groups according to the risk score of the signature, and it was found that the survival time of patients in the high-risk group was significantly shorter than that in the low-risk group (Fig. S1A and B). We then downloaded the MSI status data from the TCIA database (https://tcia.at/home). MSI-H patients had significantly higher risk scores than MSS patients in the TCGA dataset (Fig. S1C and D). Moreover, we discovered that the high-risk group was more likely to respond to immunotherapy than the low-risk group, because the expression of PD-1 and PD-L1 was significantly higher in the high-risk group than in the low-risk group (Fig. S1E and F). We also calculated TMB scores based on the TGCA somatic mutation data. The TMB in the low-risk group and high-risk group had no significant difference (Fig. S1G).

### Establishment and assessment of the nomogram based on the thirteen-IRGs signature

**Fig. 7:**
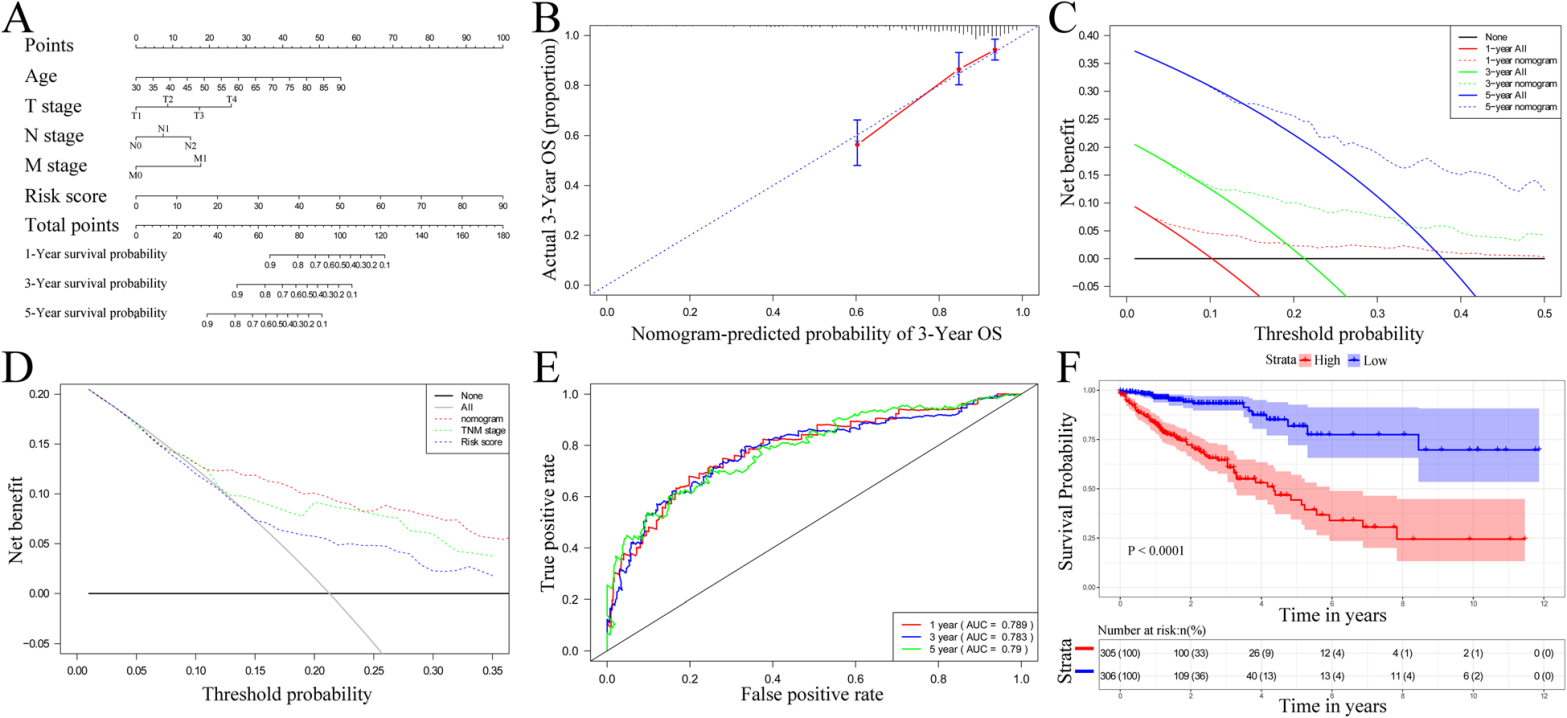
Establishment of the nomogram predicting overall survival for CRC patients in the TCGA cohort. **A**, Nomogram predicting 1-year, 3-year and 5-year OS for CRC patients. **B**, Calibration plot for nomogram predicted and observed 3-year overall survival rate. **C**, Decision curve analysis for 1-year, 3-year and 5-year overall survival predictions. **D**, Decision curve analysis comparing nomogram with the TNM stage and the thirteen-IRGs signature. **E**, Time-dependent ROC curves for overall survival prediction by the nomogram in TCGA cohort. **F**, Kaplan–Meier curves for overall survival prediction by the nomogram in TCGA cohort

A nomogram was established in terms of the smallest Akaike Information Criterion (AIC) value occurred from the multivariate Cox regression (AIC = 1132.81), which includes age, T stage, N stage, M stage and the thirteen-IRGs signature (Fig. 7A). The calibration plot demonstrated that the evaluating capability of the nomogram was best in forecasting 3-year OS (Fig. 7B). The C-index of the nomogram was 0.770 (95% CI = 0.718–0.823). As Fig. 7C displays, the nomogram illustrated a greater net benefit that had a wider range of threshold probability on the DCA for predicting 1-, 3-, and 5- year OS. Combining thirteen-IRGs signature with TNM stage showed better net benefit for predicting 3-year OS (Fig. 7D). The AUC for 1-, 3-, and 5- year OS of the nomogram in TCGA dataset was 0.789, 0.783 and 0.790, respectively (Fig. 7E). Based on Fig. 7F, CRC patients from the high-risk group, stratified by the median of nomogram ‘s risk score, had significantly lower OS rates compared to the low-risk group (*P* < 0.001). Using the GSE39582 cohort for validation, we obtained similar results, as displayed by Fig. S2.

### Survival analysis of IRGs

**Fig. 8:**
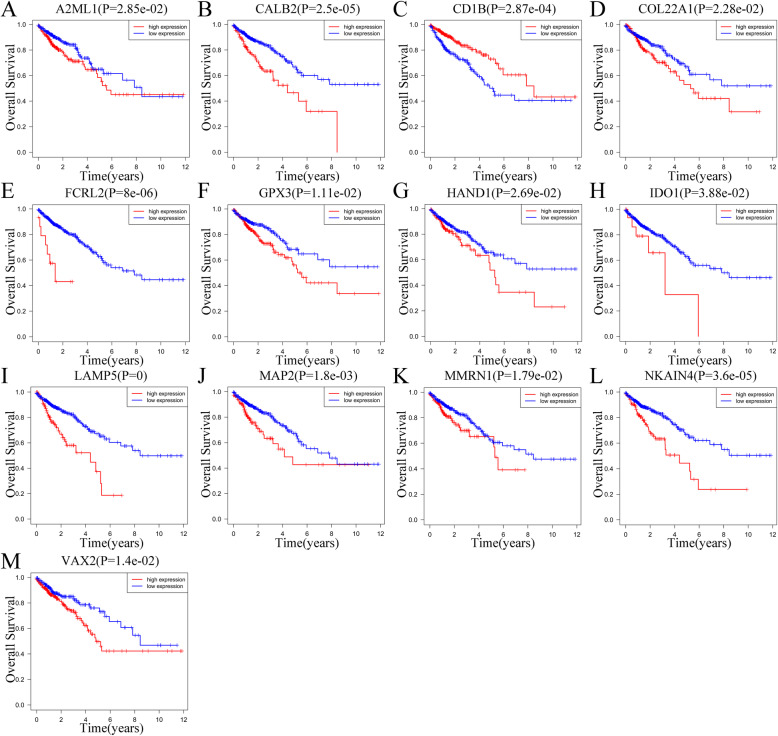
Correlation of expression of those thirteen IRGs in overall survival in TCGA. Kaplan-Meier survival curves were generated for selected immune-related genes extracted from the comparison of groups of high (red line) and low (blue line) gene expression. *p* < 0.05 in Log-rank test

For additional confirmation of their prognostic value and expression, we applied the Kaplan-Meier survival analysis for the thirteen IRGs in signature in patients with CRC from TCGA (Fig. 8). We found that A2ML1, CALB2, COL22A1, FCRL2, GPX3, HAND1, IDO1, LAMP5, MAP2, MMRN1, NKAIN4 and VAX2 were identified as cancer-promoting factors given their high expression correlation with shorter OS in patients with CRC. On the other hand, the high expression of CD1B exhibited a significant correlation with longer OS. This association implies the possible protective role of RNAs in CRC. Additionally, we verified ten IRGs have significant correlation with OS in the GSE39582 cohort, including A2ML1, CALB2, COL22A1, GPX3, HAND1, IDO1, LAMP5, MAP2, MMRN1 and NKAIN4. (Fig. S3).

### Correlation between TIICs and prognostic IRGs

**Fig. 9:**
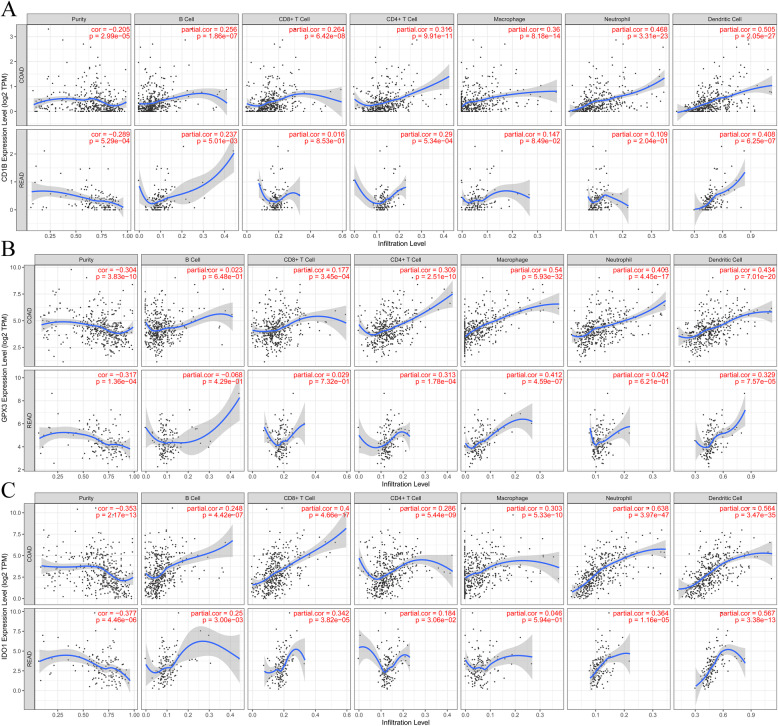
Integrative analysis between the IRGs with tumor infiltration immune cells in CRC. **A**, The correlation between CD1B gene expression and immune cells. **B**, The correlation between GPX3 gene expression and immune cells. **C**, The correlation between IDO1 gene expression and immune cells

The independent predicted factors of sentinel lymph node status and OS among cancer patients are the Tumor-infiltrating immune cells (TIICs) [34]. As a result, we explored the correlation of those thirteen prognostic IRGs with the immune infiltration levels in CRC using TIMER (Table 1). The outcomes reveal the positive correlation of the CD1B expression level with the infiltrating levels of neutrophils (*r* = 0.468, *p* = 3.31E-23) and Dendritic Cells (*r* = 0.505, *P* = 2.05E-27) in colon adenocarcinoma (COAD) (Fig. 9A). Moreover, GPX3 has the highest significant correlations with infiltrating levels of macrophages (*r* = 0.54, *p* = 5.93E-32), neutrophils (*r* = 0.408, *p* = 4.45E-17) and Dendritic Cells (*r* = 0.434, *P* = 7.01E-20) (Fig. 9B). In addition, IDO1 expression was significantly associated with infiltrating levels of CD8+ T cells (*r* = 0.4, *P* = 4.66E-17), neutrophils (*r* = 0.638, *p* = 3.97E-47) and Dendritic Cells (*r* = 0.564, *P* = 3.47E-35) (Fig. 9C).

**Table 1:** Correlation of the thirteen prognostic IRGs with the levels of immune infiltration in CRC

		Purity		B Cell		CD8+ T Cell		CD4+ T Cell		Macrophage		Neutrophil		Dendritic Cell	
		Cor	P	Cor	P	Cor	P	Cor	P	Cor	P	Cor	P	Cor	P
A2ML1	**COAD**	−0.156	0.002	−0.103	0.039	−0.016	0.743	0.136	0.006	0.048	0.339	0.019	0.705	0.025	0.624
	**READ**	−0.029	0.73	− 0.04	0.639	− 0.119	0.162	− 0.101	0.239	0.014	0.866	−0.005	0.957	0.115	0.179
CALB2	**COAD**	−0.291	2.26E-09	0.014	0.769	0.109	0.028	0.263	9.11E-08	0.331	8.51E-12	0.284	7.19E-09	0.299	8.76E-10
	**READ**	−0.334	5.49E-05	− 0.081	0.345	− 0.002	0.984	0.286	6.37E-04	0.321	1.16E-04	0.204	0.02	0.291	5.13E-04
CD1B	**COAD**	−0.205	2.99E-05	0.256	1.86E-07	0.264	6.42E-08	0.315	9.91E-11	0.36	8.18E-14	0.468	3.31E-23	0.505	2.05E-27
	**READ**	−0.289	5.29E-04	0.237	0.005	0.016	0.853	0.29	5.34E-04	0.147	0.085	0.109	0.204	0.408	6.25E-07
COL22A1	**COAD**	−0.265	5.40E-08	0.022	0.664	0.131	0.008	0.396	1.62E-16	0.473	6.29E-24	0.398	1.06E-16	0.395	1.91E-16
	**READ**	−0.296	3.84E-04	0.099	0.242	0.0006	0.994	0.411	5.07E-07	0.399	1.16E-06	0.254	0.002	0.299	3.50E-04
FCRL2	**COAD**	−0.395	1.21E-16	0.484	3.74E-25	0.237	1.35E-06	0.447	3.63E-21	0.297	1.12E-09	0.378	4.34E-15	0.434	6.73E-20
	**READ**	−0.411	4.66E-07	0.516	8.06E-11	0.232	0.006	0.123	0.15	−0.015	0.861	0.115	0.181	0.304	2.73E-04
HAND1	**COAD**	−0.176	3.49E-04	−0.103	0.038	0.038	0.446	0.256	2.06E-07	0.377	4.04E-15	0.155	0.002	0.211	1.93E-05
	**READ**	−0.067	0.433	− 0.016	0.856	− 0.06	0.479	0.219	0.01	0.317	1.46E-04	−0.019	0.828	0.111	0.191
GPX3	**COAD**	−0.3	3.83E-10	0.022	0.648	0.177	3.45E-04	0.309	2.51E-10	0.54	5.93E-32	0.402	4.45E-17	0.434	7.01E-20
	**READ**	−0.317	1.36E-04	−0.068	0.429	0.029	0.732	0.313	1.78E-04	0.412	4.59E-07	0.042	0.621	0.329	7.57E-05
IDO1	**COAD**	−0.353	2.17E-13	0.248	4.42E-07	0.4	4.66E-17	0.286	5.44E-09	0.303	5.33E-10	0.638	3.97E-47	0.564	3.47E-35
	**READ**	−0.377	4.46E-06	0.25	0.003	0.342	3.82E-05	0.184	0.031	0.046	0.594	0.364	1.16E-05	0.567	3.38E-13
LAMP5	**COAD**	−0.315	6.93E-11	0.099	0.047	0.155	0.002	0.447	3.90E-21	0.567	9.59E-36	0.351	4.22E-13	0.451	1.32E-21
	**READ**	−0.29	5.01E-04	0.184	0.03	0.084	0.325	0.331	6.73E-05	0.482	1.84E-09	0.146	0.087	0.434	9.32E-08
MAP2	**COAD**	−0.255	1.82E-07	0.125	0.012	0.286	4.59E-09	0.441	1.38E-20	0.535	3.03E-31	0.429	2.22E-19	0.465	5.68E-23
	**READ**	−0.283	7.15E-04	0.074	0.389	0.311	1.92E-04	0.214	0.012	0.419	2.74E-07	0.234	0.006	0.335	5.54E-05
MMRN1	**COAD**	−0.395	1.09E-16	0.244	7.27E-07	0.315	8.80E-11	0.482	9.52E-25	0.583	4.47E-38	0.488	2.29E-25	0.533	7.25E-31
	**READ**	−0.364	9.73E-06	0.274	0.0011	0.313	1.72E-04	0.15	0.078	0.292	4.8E-04	0.256	0.002	0.36	1.37E-05
NKAIN4	**COAD**	−0.274	1.92E-08	−0.07	0.161	−0.007	0.887	0.301	7.29E-10	0.341	1.90E-12	0.229	3.78E-06	0.273	2.79E-08
	**READ**	−0.325	9.17E-05	−0.08	0.327	−0.123	0.149	0.389	2.20E-06	0.454	1.93E-08	0.113	0.188	0.197	0.02
VAX2	**COAD**	−0.239	1.12E-06	−0.016	0.742	0.118	0.017	0.239	1.21E-06	0.352	3.26E-13	0.284	7.00E-09	0.285	5.61E-09
	**READ**	−0.108	0.203	− 0.157	0.07	− 0.119	0.163	0.206	0.015	0.139	0.104	0.012	0.89	0.096	0.261

We also found that CD1B, FCRL2, IDO1 and MMRN1 were correlated with the infiltration of B cells. Moreover, CD1B, FCRL2, GPX3, IDO1, MAP2 and MMRN1 were correlated with the infiltration of CD8+ T cells. The outcomes reveal the positive correlation of the CD1B, CALB2, COL22A1, FCRL2, GPX3, HAND1, IDO1, LAMP5, MAP2, MMRN1, NKAIN4 and VAX2 expression level with the infiltrating levels of CD4+ T cells, macrophages and Dendritic Cells. In addition, CD1B, CALB2, COL22A1, FCRL2, GPX3, IDO1, LAMP5, MAP2, MMRN1, NKAIN4 and VAX2 expression was significantly associated with infiltrating levels of neutrophils.

## Discussion

The TME affects the progression and growth of a tumor [35]. Moreover, it is comprised of the tumor and non-tumor cells like fibroblasts and immune cells [36]. Tumor-infiltrating immune cells are significantly linked to oncogenesis and angiogenesis, as well as to the metastasis and growth of tumor cells, which could regulate the abundance and differentiation of immune cells [37]. Recent literature highlights how the discrepancies between tumor progression and host’s immune response could result in tumor’s growth [38]. Thus, it is vital to understand the stromal and immune status in the TME to develop strategies that could enhance the patient’s response rate to immunotherapy.

The cutting-edge therapeutic methods include immunotherapies and molecularly targeted therapies, as well as radiotherapies and chemotherapies. However, limitations remain prevalent and include toxicity, immunity of patients to treatments, low response rates, and financial burdens caused by high costs [39, 40]. Also, modern cancer treatments still need biomarkers for prognosis. Hence, we attempted to find prognostic IRGs that impact the patients’ OS by analyzing the TME.

Some studies applied the ESTIMATE to some types of cancers [19, 20], which signify the benefits of using algorithms in large data sets. For example, Vincent et al. used this algorithm to compute for the breast cancer tumor purity and prove that the immune and stromal components are nonexistent in vitro [41]. To comprehend the TME of CRC, we applied the ESTIMATE to generate tumor purity, as well as the stromal and immune scores. In recent studies, [42, 43] our analysis deduced the positive correlation of stromal scores and immune scores with the stages of a tumor, and tumor purity was inversely correlated with tumor stages. Moreover, longer survival times highlighted the presence of low tumor purity and high stromal and immune scores amongst the CRC patients. This suggests the key role of TME in patient outcomes.

We classified the TCGA patients into high and low stromal scores, immune scores and tumor purity groups to detect IRGs. Then, we applied the GO analysis to confirm the involvement of those genes in the TME, such as leukocyte activation, leukocyte migration, T cell activation, extracellular matrix, side of membrane and receptor regulator activity. The outcomes have been consistent with the results of previous studies, where immune cells and ECM molecules influenced the creation of the interdependence within the CRC’s tumor microenvironment [14, 35]. Also, we established the PPI modules to unveil the association and purpose of IRGs. The nodes with high degree of connectivity among modules, such as C3, FBN1 and ITGB2, were linked to apoptosis, migration, angiogenesis, proliferation, and immune response [44–46].

Then, we performed LASSO Cox analyses to generate a thirteen-IRGs signature for predicting the prognosis of OS patients in TCGA dataset, and cross-validated from the GEO database GSE39582. The AUC values for the signature in predicting 1, 3, and 5-year survival were 0.703, 0.711, and 0.676, which indicates a good capability for predicting survival in CRC patients. We further evaluated the association between the signature and clinical parameters to figure out the clinical value of this thirteen-IRGs signature. It was found that high-risk patients were associated significantly with TNM stages. DCA and ROC analysis indicated that the nomogram consists of the thirteen-IRGs signature and conventional clinical parameters exhibited high performance in prognostic sensitivity for patients with CRC. These results demonstrated great applicability and stability of the thirteen-IRGs signature for predicting prognosis of patients with CRC.

To ascertain the association of IRGs with immune infiltration, we used a marker called CD1B. Guo et al. found that miR-582/CD1B, which are involved in resting and activated dendritic cells, may be potential novel biomarkers for immunotherapy [47]. Similar to our study, in prostate cancer, the decreased levels of CD1B expression could be linked to the poorer disease-free survival [48]. Finally, using the TIMER algorithm, we evaluated how prognosis-related genes are correlated with immune-infiltrating levels in CRC. Our results suggest that in both READ and COAD, the expression level of CD1B is significantly positive correlation with the tumor-infiltrating levels of neutrophils and Dendritic Cells.

Previous studies indicated that IDO1 suppressed the CD8+ T cell response in colon cancer, which may provide a theoretical basis for the development of new immunotherapy for the treatment of colon cancer [49]. The positive correlation of the IDO1 expression with PD-L1 pathways on T cells is also evident [50]. Furthermore, during the PD-L1 therapy, both CD8^+^ and CD4^+^ T cells had higher abundance levels in the TME. As a result, this activates the IDO1 on the T cells, which improves this process and causes improvements in tumor immunity.

To our understanding, this is a pioneering study on CRC that investigates IRGs in the TME. Thus, we took advantage of the opportunities. First, we used the GEO and TCGA databases to study the TME. Both sources gave us an extensive amount of CRC samples. We crosschecked our results using independent cohorts. Then, we acknowledged the complexities associated with the TME, which include genetic factors among many others. Furthermore, this study extensively evaluated how the TME (immune score, stromal scores and tumor purity) interacted with prognostic IRGs.

## Conclusions

In conclusion, our current study established a thirteen-gene signature based on ESTIMATE algorithm-derived immune/stromal scores, which could serve as a favorable prognostic factor. Our model would be of clinical value and provide additional data for better understanding of the TME. Finally, the results of functional analysis provide a novel insight for revealing the molecular mechanism in tumor initiation and progression.

## Supplementary Information

**Additional file 1: Table S1.** Top 20 KEEG pathways and GO terms enriched by the DEmRNAs.

**Additional file 2: Figure S1.** Estimation of the MSI status and cancer immunotherapy response using the thirteen-IRGs signature in the TCGA cohort.

**Additional file 3: Figure S2.** Validation of the nomogram predicting overall survival for CRC patients in the GSE39582 cohort.

**Additional file 4: Figure S3.** Validation of correlation of IRGs extracted from TCGA database with overall survival in GEO cohort. Kaplan-Meier survival curves were generated for selected immune-related genes extracted from the comparison of groups of high (red line) and low (blue line) gene expression. *p* < 0.05 in Log-rank test.

## Data Availability

The data that support the findings of this study are available in The Cancer Genome Atlas (TCGA) and Gene Expression Omnibus (GEO). These data were derived from the following resources available in the public domain: [https://tcga-data.nci.nih.gov/] and [https://www.ncbi.nlm.nih.gov/geo/].

## Abbreviations

TME: Tumor microenvironment
IRGs: Immune-related genes
CRC: Colorectal cancer
TCGA: The Cancer Genome Atlas
GEO: Gene Expression Omnibus
ESTIMATE: Estimation of STromal and Immune cells in MAlignant Tumor tissues using Expression data
DEGs: Differentially expressed genes
PPI: Protein-protein interaction
LASSO: Least absolute shrinkage and selection operator
OS: Overall survival
TIICs: Tumor-infiltrating immune cells
FDR: False discovery rate
GO: Gene ontology
MF: Molecular functions
BP: Biological processes
CC: Cellular components
KEGG: Kyoto Encyclopedia of Genes and Genomes
ROC: Receiver operating characteristic
DCA: Decision curve analysis
AIC: Akaike Information Criterion
