# Supplementary material for: Identification of prognostic immune-related gene signature associated with tumor microenvironment of colorectal cancer
Source: BMC Cancer. 2021 Aug 8;21:905. doi: 10.1186/s12885-021-08629-3 (PMC8349485; doi:10.1186/s12885-021-08629-3)
Supplement: Supplementary file 1 — Additional file 1: Table S1. Top 20 KEEG pathways and GO terms enriched by the DEmRNAs. [file 12885_2021_8629_MOESM1_ESM.docx]

**Table S1: Top 20 KEEG pathways and GO terms enriched by the DEmRNAs**

| Categories | Term | Description | Count | FDR |
| --- | --- | --- | --- | --- |
| KEGG pathways | hsa04060 | Cytokine-cytokine receptor interaction | 68 | 7.07E-21 |
|  | hsa04062 | Chemokine signaling pathway | 45 | 1.87E-14 |
|  | hsa04151 | PI3K-Akt signaling pathway | 42 | 0.00015 |
|  | hsa04145 | Phagosome | 41 | 4.66E-15 |
|  | hsa04061 | Viral protein interaction with cytokine and cytokine receptor | 40 | 5.47E-21 |
|  | hsa04514 | Cell adhesion molecules | 40 | 8.91E-15 |
|  | hsa04640 | Hematopoietic cell lineage | 39 | 1.29E-20 |
|  | hsa05152 | Tuberculosis | 39 | 3.10E-11 |
|  | hsa05150 | Staphylococcus aureus infection | 38 | 2.82E-20 |
|  | hsa04380 | Osteoclast differentiation | 35 | 3.77E-13 |
|  | hsa05323 | Rheumatoid arthritis | 33 | 6.79E-16 |
|  | hsa04080 | Neuroactive ligand-receptor interaction | 32 | 0.044187 |
|  | hsa05322 | Systemic lupus erythematosus | 27 | 4.96E-07 |
|  | hsa05140 | Leishmaniasis | 26 | 3.65E-12 |
|  | hsa05166 | Human T-cell leukemia virus 1 infection | 25 | 0.010256 |
|  | hsa04015 | Rap1 signaling pathway | 24 | 0.012142 |
|  | hsa04610 | Complement and coagulation cascades | 23 | 1.18E-08 |
|  | hsa04620 | Toll-like receptor signaling pathway | 23 | 6.13E-07 |
|  | hsa05164 | Influenza A | 23 | 0.001841 |
|  | hsa04510 | Focal adhesion | 23 | 0.014064 |
| GO BP | GO:0002694 | regulation of leukocyte activation | 103 | 6.30E-35 |
|  | GO:0050900 | leukocyte migration | 99 | 3.21E-34 |
|  | GO:0042110 | T cell activation | 89 | 4.20E-29 |
|  | GO:0001819 | positive regulation of cytokine production | 87 | 1.47E-27 |
|  | GO:0002521 | leukocyte differentiation | 87 | 5.98E-25 |
|  | GO:0042119 | neutrophil activation | 86 | 1.43E-23 |
|  | GO:0043062 | extracellular structure organization | 85 | 1.93E-30 |
|  | GO:0050727 | regulation of inflammatory response | 85 | 4.19E-24 |
|  | GO:0002446 | neutrophil mediated immunity | 83 | 1.04E-21 |
|  | GO:0002283 | neutrophil activation involved in immune response | 82 | 1.03E-21 |
|  | GO:0043312 | neutrophil degranulation | 81 | 2.58E-21 |
|  | GO:0030198 | extracellular matrix organization | 80 | 3.30E-31 |
|  | GO:0051249 | regulation of lymphocyte activation | 79 | 3.04E-25 |
|  | GO:0050867 | positive regulation of cell activation | 77 | 2.08E-30 |
|  | GO:0032103 | positive regulation of response to external stimulus | 75 | 3.29E-29 |
|  | GO:0007159 | leukocyte cell-cell adhesion | 75 | 4.35E-28 |
|  | GO:0022407 | regulation of cell-cell adhesion | 74 | 8.00E-23 |
|  | GO:0002683 | negative regulation of immune system process | 74 | 4.99E-19 |
|  | GO:0002696 | positive regulation of leukocyte activation | 72 | 7.11E-28 |
|  | GO:0045785 | positive regulation of cell adhesion | 71 | 6.54E-21 |
| GO CC | GO:0031012 | extracellular matrix | 108 | 7.05E-42 |
|  | GO:0098552 | side of membrane | 63 | 1.13E-17 |
|  | GO:0030667 | secretory granule membrane | 60 | 5.51E-20 |
|  | GO:0009897 | external side of plasma membrane | 49 | 6.66E-20 |
|  | GO:0098797 | plasma membrane protein complex | 49 | 1.62E-06 |
|  | GO:0070820 | tertiary granule | 40 | 3.40E-16 |
|  | GO:0005788 | endoplasmic reticulum lumen | 39 | 6.82E-07 |
|  | GO:0045121 | membrane raft | 39 | 8.33E-07 |
|  | GO:0098857 | membrane microdomain | 39 | 8.54E-07 |
|  | GO:0098589 | membrane region | 39 | 1.85E-06 |
|  | GO:0060205 | cytoplasmic vesicle lumen | 38 | 1.33E-05 |
|  | GO:0031983 | vesicle lumen | 38 | 1.38E-05 |
|  | GO:0030139 | endocytic vesicle | 37 | 1.14E-06 |
|  | GO:0034774 | secretory granule lumen | 37 | 1.11E-05 |
|  | GO:0043025 | neuronal cell body | 37 | 0.008267 |
|  | GO:0042581 | specific granule | 36 | 1.87E-13 |
|  | GO:0043235 | receptor complex | 35 | 9.28E-06 |
|  | GO:0031252 | cell leading edge | 32 | 0.01119 |
|  | GO:0005581 | collagen trimer | 31 | 1.05E-17 |
|  | GO:0005925 | focal adhesion | 31 | 0.031037 |
| GO MF | GO:0030545 | receptor regulator activity | 60 | 5.78E-12 |
|  | GO:0048018 | receptor ligand activity | 59 | 1.08E-12 |
|  | GO:0005539 | glycosaminoglycan binding | 54 | 1.74E-23 |
|  | GO:0030246 | carbohydrate binding | 54 | 1.60E-18 |
|  | GO:0005201 | extracellular matrix structural constituent | 52 | 9.39E-27 |
|  | GO:1901681 | sulfur compound binding | 45 | 1.81E-14 |
|  | GO:0008201 | heparin binding | 42 | 5.78E-19 |
|  | GO:0005126 | cytokine receptor binding | 37 | 3.93E-10 |
|  | GO:0005125 | cytokine activity | 35 | 1.08E-12 |
|  | GO:0008047 | enzyme activator activity | 35 | 0.011512 |
|  | GO:0001664 | G-protein coupled receptor binding | 34 | 1.14E-08 |
|  | GO:0033218 | amide binding | 31 | 0.000101 |
|  | GO:0004175 | endopeptidase activity | 30 | 0.005183 |
|  | GO:0042277 | peptide binding | 29 | 1.35E-05 |
|  | GO:0003779 | actin binding | 29 | 0.043175 |
|  | GO:0019955 | cytokine binding | 25 | 3.93E-10 |
|  | GO:0005178 | integrin binding | 25 | 5.48E-09 |
|  | GO:0019838 | growth factor binding | 22 | 1.90E-06 |
|  | GO:0005518 | collagen binding | 21 | 2.67E-11 |
|  | GO:0031406 | carboxylic acid binding | 21 | 0.00166 |
