# Supplementary figures and images for: Identification of prognostic immune-related gene signature associated with tumor microenvironment of colorectal cancer
Source: BMC Cancer. 2021 Aug 8;21:905. doi: 10.1186/s12885-021-08629-3 (PMC8349485; doi:10.1186/s12885-021-08629-3)

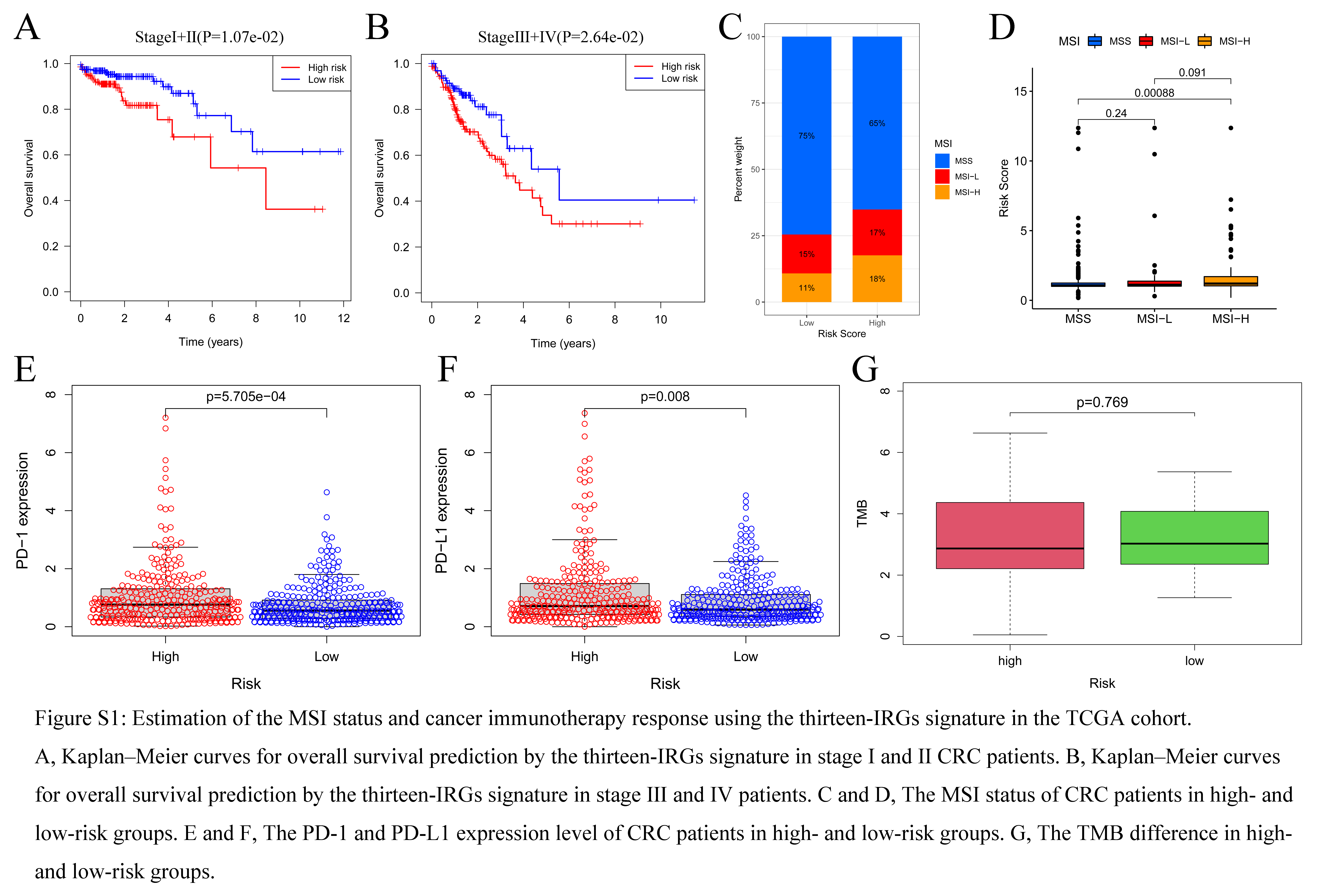

Supplement: Supplementary file 2 — Additional file 2: Figure S1. Estimation of the MSI status and cancer immunotherapy response using the thirteen-IRGs signature in the TCGA cohort. [file 12885_2021_8629_MOESM2_ESM.tif]

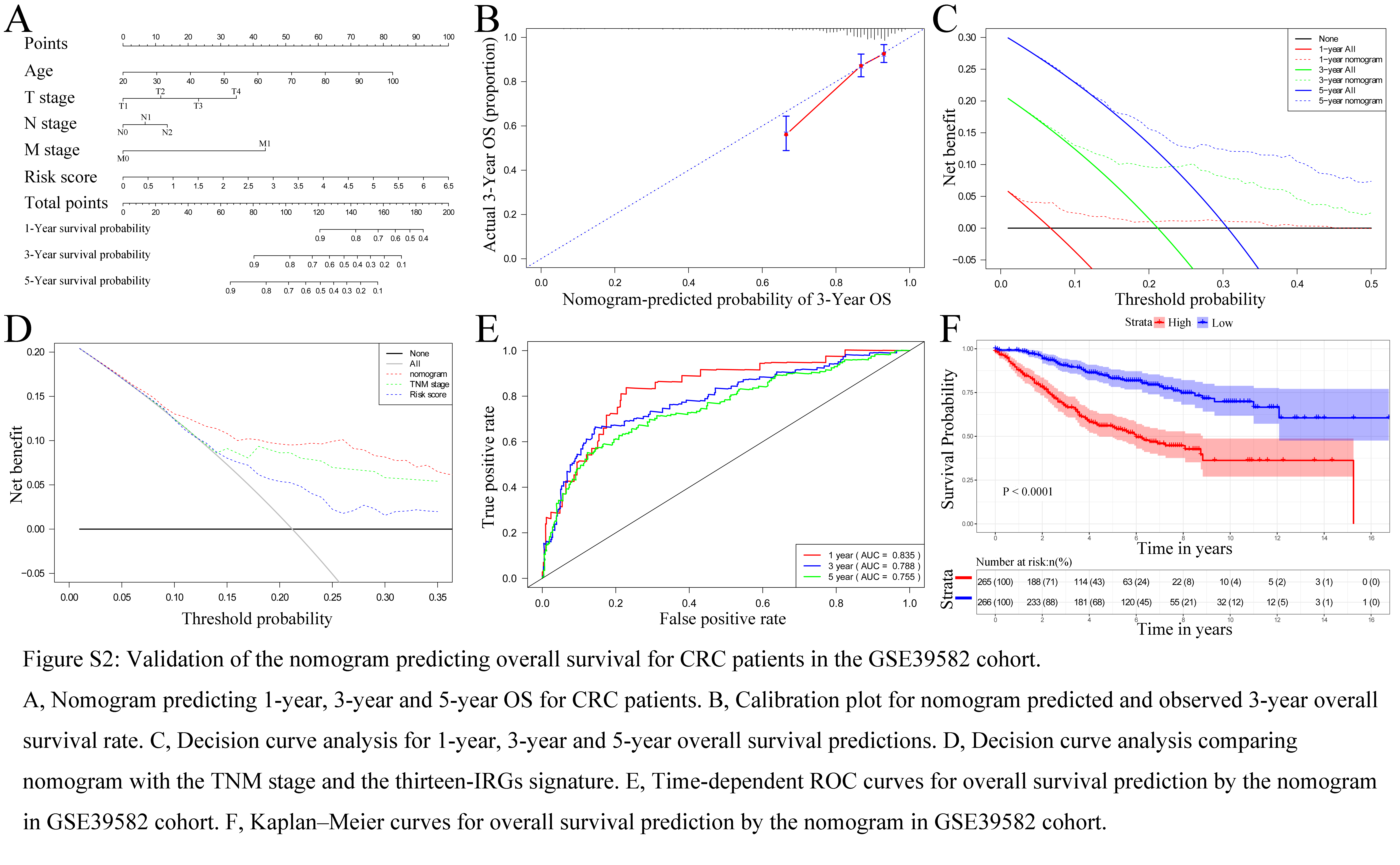

Supplement: Supplementary file 3 — Additional file 3: Figure S2. Validation of the nomogram predicting overall survival for CRC patients in the GSE39582 cohort. [file 12885_2021_8629_MOESM3_ESM.tif]
